# Predictors of Resilience Among Patients Living With Heart Failure Amid Escalating Sociopolitical Challenges: A Cross‐Sectional Approach

**DOI:** 10.1002/nop2.70816

**Published:** 2026-09-15

**Authors:** Hiba Deek, Angela R. Massouh, Wassim Shatila

**Affiliations:** ^1^ Nursing Department, Faculty of Health Sciences Beirut Arab University Beirut Lebanon; ^2^ Adult and Critical Care Nursing, Undergraduate Division American University of Beirut, Hariri School of Nursing Beirut Lebanon; ^3^ Research, Education, and Innovation American University of Beirut, Hariri School of Nursing Beirut Lebanon; ^4^ Cardiology and Heart Failure American University of Beirut, Hariri School of Nursing Beirut Lebanon; ^5^ Balamand University and Beirut Arab University Beirut Lebanon; ^6^ Clemenceau Medical Center Beirut Lebanon

**Keywords:** cardiovascular diseases, depression, quality of life, resilience, self‐management

## Abstract

**Aim:**

Exposure to chronic illness and prolonged sociopolitical adversity may shape how patients adapt to illness‐related demands; however, this relationship should be interpreted cautiously because stress can both challenge and undermine adaptive capacity. This study aimed to assess resilience and identify demographic, clinical, psychosocial, and self‐care correlates of resilience among patients living with heart failure in Lebanon.

**Design:**

A quantitative cross‐sectional observational study.

**Methods:**

Arabic‐translated and psychometrically tested instruments were used to assess depressive symptoms, self‐care, quality of life, and resilience. Data were collected between January 2024 and April 2025 from Arabic‐speaking adults with confirmed heart failure recruited through convenience sampling from outpatient clinics and primary healthcare services across several Lebanese geographic regions. Descriptive statistics, bivariate analyses, correlation analyses, and multiple linear regression were conducted. Statistical significance was set at *p* < 0.05, and reporting followed the STROBE checklist.

**Results:**

The analysis included 299 participants with a mean age of 59.95 ± 15.89 years. Almost half of the participants were older adults with a mean age of 71.18 ± 6.06 years and a slightly higher proportion of males than females. Hypertension, diabetes, and hyperlipidemia were common. Mean left ventricular ejection fraction was 37.52% ± 10.51%, and most participants were classified as New York Heart Association functional class II or III. Mean scores indicated moderate depressive symptoms, impaired HF‐related quality of life, and moderate resilience. Resilience was positively correlated with self‐care maintenance [*r* = 0.54, *p* < 0.001], self‐care monitoring [*r* = 0.47, *p* < 0.001], and self‐care management [*r* = 0.53, *p* < 0.001] and was negatively correlated with quality of life [*r* = 0.055, *p* = 0.53], although this correlation was not significant. In the multiple linear regression model, self‐care maintenance, self‐care management, gender, and loop diuretic use were significant predictors of resilience, explaining 36.1% of the variance in resilience scores. Higher self‐care maintenance and management scores and loop diuretic use were associated with higher resilience. Female gender was associated with lower resilience scores after adjustment.

**Conclusion:**

Lebanese patients living with heart failure reported moderate resilience in the context of ongoing sociopolitical and economic adversity. Higher resilience was consistently associated with stronger heart failure self‐care behaviours, particularly self‐care maintenance and management.

**Implications:**

This study addressed the limited evidence on resilience among patients living with heart failure in a context of prolonged sociopolitical and economic adversity. The main findings showed that patients had moderate resilience and that higher resilience was associated with stronger heart failure self‐care behaviors. In multivariable analysis, higher self‐care maintenance, higher self‐care management, loop diuretic use, and male gender were associated with higher resilience. The findings should guide nursing practice in caring for patients with heart failure by identifying those at high risk for faltered resilience and aim for the factors associated with this poor outcome. This applies to the Lebanese context and to similar resource‐constrained or crisis‐affected settings to assess resilience, strengthen self‐care behaviors, and provide context‐sensitive support for patients living with heart failure.

**Patient or Public Contribution:**

This study did not include patient or public involvement in its design, conduct, or reporting.

## 1 Introduction

Heart failure (HF) is a progressive clinical syndrome associated with structural or functional abnormalities of the heart that impair ventricular filling or ejection of blood. It is commonly manifested through symptoms such as dyspnea, fatigue, fluid retention, and reduced exercise tolerance, which can significantly affect daily functioning and quality of life (McDonald et al. 2021). Globally, HF affects more than 64 million people, and its prevalence increases with age, exceeding 10% among adults older than 70 years (Groenewegen et al. 2020; Brouwers et al. 2013; Meyer et al. 2015). In Lebanon and the broader Middle East and North Africa region, local epidemiological data remain limited, making it difficult to estimate the full burden of HF. However, available evidence suggests that HF care is costly in Lebanon, with hospital admissions placing substantial pressure on the healthcare system and outpatient medications often paid out‐of‐pocket by patients (Tatari et al. 2015).

Living with HF requires continuous self‐care, including symptom monitoring, medication adherence, dietary modification, fluid and salt restriction, physical activity adjustment, and timely response to worsening symptoms (Aggarwal et al. 2018; Beezer et al. 2022). These demands are central to nursing practice because nurses and advanced practice nurses play a key role in patient assessment, discharge education, self‐care counselling, medication teaching, family engagement, and follow‐up after hospitalization. In settings where access to healthcare resources is constrained, nursing‐led support becomes particularly important in helping patients recognize symptoms, maintain treatment plans, and adapt to the daily burden of illness. Nevertheless, the complexity of HF self‐care, polypharmacy, repeated healthcare encounters, and financial strain may increase psychological distress, including depression, anxiety, and stress (Angermann and Ertl 2018).

Resilience is increasingly recognized as an important adaptive factor in chronic illness. It refers to the capacity to adapt, recover, and maintain functioning in the face of adversity, but it should not be assumed to automatically increase after exposure to stress. Rather, resilience is dynamic and may be strengthened, challenged, or depleted depending on individual, social, clinical, and contextual factors (Babić et al. 2020; Manchia et al. 2022). In chronic illness populations, resilience has been associated with psychological well‐being, coping, and quality of life (Ristevska‐Dimitrovska et al. 2015; Lee et al. 2017). In HF, however, the evidence remains limited, particularly regarding how resilience relates to depressive symptoms, HF self‐care, and quality of life. Understanding these relationships is important for nursing practice because resilience may represent a modifiable domain that can be assessed and supported through education, counselling, family‐inclusive care planning, and community‐based follow‐up.

The Lebanese context provides a unique setting in which to examine resilience among patients with HF. Since 2019, Lebanon has experienced overlapping political, economic, social, and healthcare crises, including currency devaluation, inflation, rising poverty, the Beirut Port explosion, and the continuing effects of regional instability. These challenges have affected healthcare access, medication affordability, continuity of care, and patients' ability to prioritize chronic illness management. For patients with HF, such conditions may intensify the difficulty of maintaining self‐care behaviours, adhering to prescribed medications, attending follow‐up visits, and securing basic needs. Therefore, the relationship between adversity and resilience in this population cannot be assumed; it must be empirically examined.

Previous Lebanese studies have reported changes in quality of life and self‐care patterns among patients with HF over time (Deek and Massouh 2024; Zahwe et al. 2020). However, these findings do not explain whether patients' adaptation is supported by resilience or whether resilience itself is being compromised by prolonged adversity. Existing resilience studies in Lebanon have mainly focused on health system resilience or resilience among specific professional groups during crisis conditions (Alameddine et al. 2021; Ammar et al. 2016). To date, limited evidence has examined resilience among patients living with chronic illness, particularly HF, in a context of prolonged sociopolitical and economic instability. This represents an important gap in nursing science and practice because resilience assessment may help identify patients who require additional psychosocial support, self‐care reinforcement, family involvement, or closer follow‐up.

Therefore, this study aimed to assess the level of resilience and identify its key predictors among patients living with HF in Lebanon, a context marked by prolonged sociopolitical and economic adversity. The study also examined the relationships between resilience, depressive symptoms, HF self‐care, and quality of life.

## 2 Materials and Methods

### 2.1 Design

Using a quantitative cross‐sectional study, data were collected using self‐administered and interview‐based, Arabic‐translated and validated questionnaires for the Lebanese society's middle‐aged and older adult population.

#### 2.1.1 Study Setting and Sampling

Patients with HF were recruited through convenience sampling from various geographic regions across the Lebanese community to ensure national representativeness and enhance the generalizability of the study findings. Patients living with heart failure were sought in outpatient clinics and primary healthcare services. Eligible participants were Arabic‐speaking individuals aged between 30 and 80 years, in order to capture HF patients earlier in their disease trajectory and have confirmed HF with reduced ejection fraction [Left Ventricular Ejection Fraction [LVEF] less than or equal to 40%] or symptomatic HF with preserved ejection fraction [echocardiographic evidence of diastolic dysfunction] for at least 3 months prior to inclusion in the study. The diagnosis was confirmed using the Framingham criteria (McKee et al. 1971) by the researcher prior to data collection. The data collectors were cardiology nurses with a master's degree who were further trained for this type of data collection and to ensure consistency in the data collection. Data collectors visited the sites three times weekly at different schedules to ensure coverage of patients at different times. The study continued until the target sample size was achieved which took place between January 2024 and October 2025. HF patients were excluded from the study if they were clinically unstable, in an acute exacerbation, had a concomitant terminal illness such as cardiogenic shock, documented dementia, documented severe psychological illness [excluding depression] or any impairment [e.g., any uncorrected visual or hearing problems] that impeded them from filling a questionnaire.

Sample size calculation was based on the primary outcome, resilience. With a confidence interval of 95%, power of 95% and an effect size of 0.205, the sample size was calculated using G*Power to be 300 participants with complete data. The effect size was taken from a study that looked at the difference between the control and the intervention group on the resilience level after an educational intervention (Seyedoshohadaee et al. 2018).

#### 2.1.2 Study Instruments

Data were collected electronically to ensure proper data handling and storage. A link was generated on Google Forms and disseminated among the research team. A structured face‐to‐face interview was used to obtain information on demographics such as gender, age, marital status, number of persons in the household, education, employment, and income. Information on the aetiology of HF, minimum LVEF value, hospital readmissions, number of years since the diagnosis, New York Heart Association [NYHA] functional class, an unweighted index of the number of comorbid medical conditions using Charlson's Comorbidity Index [CCI] (Roffman et al. 2016). Home medications were self‐reported by the patients with reference to their documented prescriptions and recorded by the researcher. A perceived adherence scale ranging from 1 (non‐adherent) to 5 (totally adherent) was also sought through self‐administration. Quality of life, depression, self‐care, and resilience were evaluated using the below measures:

##### 2.1.2.1 Minnesota Living With HF Questionnaire [MLHFQ]

This questionnaire is used to evaluate quality of life among patients living with HF. It addresses the physical and emotional aspects. It is composed of 21 items evaluated on a 6‐point Likert scale ranging between 0 [None] and 5 [Very Much]. The score ranges from 0 to 105, with higher scores indicating worse quality of life (Bilbao et al. 2016). The questionnaire has previously been translated into Arabic and validated in the Arabic setting and was shown to have good psychometric properties (Zahwe et al. 2020). The Cronbach's alpha of the MLHFQ in the current Lebanese setting was found to be 0.96.

##### 2.1.2.2 Patient Health Questionnaire‐9 [PHQ‐9]

This is a 9‐item surveillance tool that is used to evaluate depressive symptoms. The items address changes in sleep and eating, interest, tiredness, concentration, and suicidal ideation. Each item is rated on a four‐point Likert scale, with zero being not at all to three being nearly every day. The total score ranges between 0 and 27, where higher scores indicate higher severity of depression (Löwe et al. 2004). The scores of the PHQ‐9 were further subcategorized to reflect severity of the findings as follows: scores ranging between 0 and 4 indicate none to minimal depression, 5–9 indicate mild depression, 10–14 indicate moderate depression, 15–19 indicate moderately severe depression, and 20–27 indicate severe depression (Kroenke and Spitzer 2002). The PHQ‐9 has been translated to Arabic and was shown to have good psychometric properties (AlHadi et al. 2017). The ease of administration was previously established (Deek and Massouh 2024), and the Cronbach's alpha of this tool in the Lebanese setting was found to be 0.86.

##### 2.1.2.3 Revised Self‐Care **HF** Index Version 7.2 [SCHFI]

This is a 29‐item tool divided into three subscales measuring *Self*‐*Care Maintenance*, *Symptom Perception*, and *Self‐Care Management*. The Self‐Care Confidence scale [10 questions] is an addition to this version of the SCHFI (Riegel et al. 2019). The Symptom Perception (monitoring) scale includes 9 items assessing frequency of behaviours and 2 items on how quickly symptoms were recognized and identified as HF‐related. The Self‐Care Management scale includes 8 items. Seven of these items ask how likely the respondent would be to try behaviours commonly used to control HF symptoms (1 through 5; not likely to very likely). One Self‐Care Management question asks how sure the patient is that the treatment last used to manage symptoms helped with feeling better. Response options for this item range from 0 (I did not do anything) or 1 (not sure) to 5 (very sure). Each subscale is scored separately. Response choices for all items in the scale are summed and standardized to achieve a possible score of 0–100, with higher scores indicating better self‐care. Based on an earlier version of the SCHFI v6.2, a score of 70 or better can be used to identify an adequate level of HF self‐care (Riegel et al. 2019). Reliability estimates were 0.70 or greater for all scales. Predictive validity was supportive, with significant correlations between SCHFI scores and health‐related quality‐of‐life scores (Riegel et al. 2019). Cronbach's alpha values of the overall SCHFI and the maintenance, monitoring, and management subscales were 0.885, 0.891, and 0.867, respectively, in the current sample of the Lebanese community.

##### 2.1.2.4 Connor‐Davidson Resilience Scale

The CD‐RISC is a self‐report assessment composed of 25 items that evaluate resilience. The scale was developed following an understanding that certain traits in individuals can influence their ability to cope and adapt to stressors. Therefore, the items of the scale were derived from theories addressing patience, self‐confidence, and adaptability. Thus, it reflects the following five factors: (1) personal competence, high standards, and tenacity; (2) trust in one's instincts; (3) positive acceptance of change; (4) control; and (5) spiritual influences. The items of the scale are rated on a 5‐point Likert scale ranging from 0 (not at all) to 4 (true nearly all of the time). The scores range between 0 and 100, and higher scores reflect higher resilience (K. M. Connor and Davidson 2003). It was suggested by the developer that a score of ≤ 55 is suggestive of a need to enhance coping and resilience. Therefore, this cut‐off score was used in the analysis (Connor 2020). The scale had been evaluated psychometrically and was shown to have a good internal consistency, with a Cronbach's alpha of 0.89 and an item‐total correlation ranging between 0.3 and 0.7. In terms of validity, it was shown to be positively correlated with scales addressing social support and hardiness and negatively associated with scales addressing perceived stress and disability (AlHadi et al. 2017). The scale had been previously translated into Arabic and evaluated among Lebanese women in the Lebanese setting. It was shown to have adequate psychometric properties in terms of convergent and divergent validity as reported elsewhere and a Cronbach's alpha of 0.89 (Bizri et al. 2022). The Cronbach's alpha of the CD‐RISC in the current Lebanese setting was found to be 0.965.

#### 2.1.3 Data Management and Analysis Plan

Characteristics of the study sample were described using frequencies and percentages for categorical variables and means and standard deviations for continuous variables. Quantitative data were analysed using IBM SPSS Statistics version 29.0 for Windows. Normality of continuous variables was assessed using the Kolmogorov–Smirnov test and visual inspection where appropriate. For bivariate analyses, chi‐square tests were used for categorical variables, independent‐sample *t*‐tests were used to compare means across groups, and Pearson's correlation coefficient was used to examine associations between continuous variables. The level of statistical significance was set at *p* < 0.05, with 95% confidence intervals reported where applicable.

Resilience was analysed as a continuous outcome using the total Connor‐Davidson Resilience Scale (CD‐RISC) score. Multiple linear regression analysis was conducted to identify predictors of resilience. Variables entered into the model were selected based on theoretical relevance, prior literature on resilience and heart failure, and clinical importance, rather than bivariate significance alone. The final model included self‐care maintenance, self‐care management, gender, and loop diuretic use as predictors of resilience.

The final regression model reported unstandardized coefficients, standard errors, standardized coefficients, 95% confidence intervals, *p* values, *R* ^2^, and adjusted *R* ^2^. Model assumptions and diagnostics were assessed and reported, including multicollinearity using variance inflation factor (VIF) and tolerance values, residual assumptions, influential observations, and missing‐data handling. Cases with missing values on variables included in the regression model were handled using listwise deletion. The dichotomized CD‐RISC cut‐off was retained only for descriptive comparisons and was not used as the primary outcome in the multivariable model.

#### 2.1.4 Ethical Considerations

Ethical approval was secured from the Institutional Review Board of Beirut Arab University [2023‐H‐0156‐HS‐R‐0550]. This ethical committee provides independent and timely decisions based on adherence to the guidelines in compliance with international declarations, including the Nuremberg, Helsinki, and Belmont declarations. After ensuring that participants understood the study goal, a signed informed consent was secured, and data were collected by a trained research team. Data reporting adhered to the STROBE reporting checklist.

## 3 Results

### 3.1 Sample Characteristics

A total of 475 participants were screened for enrolment in the study. However, after investigating their understanding of the term heart failure, only 345 were enrolled. The term was found to be linked to misinterpretation of heart failure among those who had undergone open heart surgeries. Additionally, after removing those with missing variables, a total of 299 participants were analysed. The mean age of the participants in this sample was 59.95 ± 15.89 years with more male participants than females participants (57.2 vs. 42.8 respectively). Of these, 147 participants were aged more than 64 years (49.3%) with a mean age of 71.18 ± 6.06 years. Two‐thirds were married and Lebanese with almost half living in the capital city. One fifth had no education and almost all the participants had a family caregiver. The majority were unemployed with almost half having a monthly income of $200–500. Details about the participants' sociodemographic status are presented in Table 1.

**TABLE 1:** Sociodemographic characteristics of the study participants (*N* = 299).

Variables	Total (*N* = 299, 100%)	Resilient (*n* = 153, 51.2%)	Non‐resilient (*n* = 146, 48.8)	*p* value (CI)
Age*	59.95 (15.89)	59.97 (15.48)	59.94 (16.37)	0.988
Gender				
Male	171 (57.2)	92 (53.8)	79 (46.2)	0.29
Female	128 (42.8)	61 (47.7)	67 (52.3)	
Social status				
Single	14 (4.7)	5 (3.3)	9 (6.2)	0.36
Married	198 (66.2)	104 (68)	94 (64.4)	
Divorced	28 (9.4)	17 (11.1)	11 (7.5)	
Widowed	59 (19.7)	27 (17.6)	32 (21.9)	
Nationality				
Lebanese	172 (57.5)	89 (58.2)	83 (56.8)	0.08
Palestinian	90 (30.1)	45 (29.4)	45 (30.8)	
Syrian	28 (9.4)	11 (7.2)	17 (11.6)	
Iraqi	2 (0.7)	1 (0.7)	1 (0.7)	
Other	7 (2.3)	7 (4.6)	0 (0)	
Living region				
Beirut	143 (47.8)	68 (44.4)	75 (51.4)	0.56
Mount Lebanon	66 (22.1)	37 (24.2)	29 (19.9)	
South Lebanon	59 (19.7)	31 (20.3)	28 (19.2)	
Beqaa Region	19 (6.4)	12 (7.8)	7 (4.8)	
North Lebanon	12 (4)	5 (3.3)	7 (4.8)	
Educational status				
No education	60 (20.1)	25 (16.3)	35 (24)	0.00
Primary education	76 (25.4)	28 (18.3)	48 (32.9)	
Secondary education	97 (32.4)	54 (35.3)	43 (29.5)	
Higher education	66 (22.1)	46 (30.1)	20 (13.7)	
Smoker	190 (63.5)	88 (57.5)	102 (69.9)	0.02** (0.362, 0.941)
Alcohol consumption	50 (16.7)	26 (17)	24 (16.4)	0.89
Flu vaccine	138 (46.2)	94 (38.6)	102 (69.9)	0.00** (2.28, 5.97)
COVID vaccine	240 (80.3)	139 (57.9)	101 (42.1)	0.00** (2.30, 8.49)
Family caregiver				
Spouse	146 (49)	85 (55.6)	61 (42.1)	0.02**
Child	130 (43.6)	57 (37.3)	73 (50.3)	
Sibling	9 (3)	2 (1.3)	7 (4.8)	
Parent	6 (2)	3 (2)	3 (2)	
Niece/Nephew	3 (1)	3 (2)	0 (0)	
No one	4 (1.3)	3 (2)	1 (0.7)	
Occupation				
Unemployed	220 (73.6)	119 (77.8)	101 (69.2)	0.22
Employed	76 (25.4)	33 (21.6)	43 (29.5)	
Self‐employed	3 (1)	1 (0.7)	2 (1.4)	
Income				
< $200	73 (24.4)	39 (25.5)	34 (23.3)	0.05
$200–$500	140 (46.8)	62 (40.5)	78 (53.4)	
$500–$1000	70 (23.4)	40 (26.1)	30 (20.5)	
> $1000	16 (5.4)	12 (7.8)	4 (2.7)	

Abbreviations: CI: confidence interval; COVID, coronavirus diseases.

* data presented in means and standard deviation.

** significant at less than 0.05; *p* value calculated using Pearson Chi‐square test.

In terms of the medical profile, more than 90% of the participants in this sample were hypertensive, more than 60% had diabetes and a similar percentage were hyperlipidemic. Psychological conditions were also evident with 35.5% diagnosed with depression. The mean CCI was 5.58 ± 2.32. When looking at the HF profile, the mean LVEF was 37.52% ± 10.51% and the majority were classified as NYHA functional class III [44.5%] followed by class II [37.8%] given that this is a community sample. The participants had been living with HF for a mean of 5.30 ± 3.49 years. In terms of medication management, 73.7% were taking angiotensin‐converting enzyme inhibitors [ACEIs] or angiotensin receptor blocking agents [ARBs], a similar number were taking loop diuretics, 90.8% were taking beta blockers and 41.3% were taking calcium channel blockers [CCBs]. In terms of medication adherence, more than one third [40.5%] perceived themselves as totally adherent as presented in Table 2.

**TABLE 2:** Medical profile of the study participants (*N* = 299).

Variables	Total (*N* = 299, 100%)	Resilient (*n* = 153, 51.2%)	Non‐resilient (*n* = 146, 48.8)	*p*
Medical profile				
CVD	56 (18.7)	25 (44.6)	31 (55.4)	0.27
COPD	96 (32.1)	47 (49)	49 (51)	0.59
Dementia	14 (4.7)	1 (7.1)	13 (92.9)	0.001** (0.009, 0.52)
Depression	106 (35.5)	53 (50)	53 (50)	0.76
Diabetes without EOD	184 (61.5)	97 (52.7)	87 (47.3)	0.49
Diabetes with EOD	19 (6.4)	7 (36.8)	12 (63.2)	0.19
Hypertension	270 (90.3)	144 (53.3)	126 (46.7)	0.02** (1.11, 5.77)
Liver disease (Mild)	26 (8.7)	9 (34.6)	17 (65.4)	0.07
Liver disease (moderate/severe)	8 (2.7)	3 (37.5)	5 (62.5)	0.43
Renal disease	46 (15.4)	21 (45.7)	25 (54.3)	0.41
Tumour	16 (5.4)	5 (31.3)	11 (68.8)	0.10
MI	128 (42.8)	73 (57)	55 (43)	0.07
PVD	46 (15.4)	24 (15.7)	22 (15.1)	0.88
Ulcer disease	44 (14.7)	20 (45.5)	24 (54.5)	0.41
Hemiplegia	8 (2.7)	3 (37.5)	5 (62.5)	0.43
Skin ulcers	28 (9.4)	12 (42.9)	16 (57.1)	0.35
On warfarin	75 (25.1)	42 (56)	33 (44)	0.33
Leukaemia	7 (2.3)	1 (14.3)	6 (85.7)	0.04** (0.018, 1.29)
Lymphoma	5 (1.7)	1 (20)	4 (80)	0.16
Metastatic solid tumour	4 (1.3)	2 (50)	2 (50)	0.96
HIV/AIDs	11 (3.7)	3 (27.3)	8 (72.7)	0.10
Hyperlipidemia	173 (57.9)	99 (57.2)	74 (42.8)	0.014** (1.12, 2.83)
CCI*	5.58 (2.32)	5.52 (1.94)	5.64 (2.68)	0.65
Medical follow up before COVID	244 (81.6)	131 (85.6)	113 (37.8)	0.06
Current medical follow up	260 (87)	144 (48.2)	116 (38.8)	0.000** (1.88, 9.06)
Surgical history	178 (59.5)	100 (56.2)	78 (43.8)	0.036** (1.03, 2.62)
Ejection fraction*	37.52 (10.51)	38.06 (10.76)	36.93 (10.24)	0.363
NYHA Class				
I	8 (2.7)	5 (3.3)	3 (2.1)	0.05
II	113 (37.8)	50 (32.7)	63 (43.2)	
III	133 (44.5)	79 (51.6)^a^	54 (37)^b^	
IV	45 (15.1)	19 (12.4)	26 (17.8)	
Years since diagnosis*	5.30 (3.49)	5.16 (2.81)	5.45 (4.07)	0.48
Medical treatment				
ACEI/ARB	213 (73.7)	122 (57.3)	91 (42.7)	0.001** (1.41, 4.18)
Beta blockers	268 (90.8)	142 (53)	126 (47)	0.052
Loop diuretics	222 (74.2)	126 (56.8)	96 (43.2)	0.001** (1.43, 4.40)
Potassium sparing diuretics	102 (34.1)	57 (55.9)	45 (44.1)	0.41
Other diuretics	60 (20.1)	26 (43.3)	34 (56.7)	0.08
Inotropes	53 (17.7)	29 (54.7)	24 (45.3)	0.65
Calcium channel blockers	121 (41.3)	60 (49.6)	61 (50.4)	0.57
Medication adherence				
1	18 (6)	5 (3.3)	13 (8.9)	0.00**
2	6 (2)	2 (1.3)	4 (2.7)	
3	51 (17.1)	10 (6.5)^a^	41 (28.1)^b^	
4	103 (34.4)	58 (37.9)	45 (30.8)	
5	121 (40.5)	78 (51)^a^	43 (29.5)^b^	

Abbreviations: ACEI/ARBs, angiotensin converting enzyme inhibitors/Angiotensin receptor blocker; CCI, charlson''s comorbidity index; COPD, chronic obstructive pulmonary disease; COVID, coronavirus disease; CVD, cerebrovascular diseases; EOD, end organ damage; HIV/AID, human immunodeficiency virus; MI, myocardial infarction; NYHA class, New York Heart Association functional classification; PVD, peripheral vascular disease.

* data presented in means and standard deviation.

** significant at less than 0.05; *p* value calculated using Pearson Chi‐square test.

^a,b^ The significant difference lies between these variables.

### 3.2 Self‐Care, Depression and Quality of Life Scores Among the Study Participants

The scores of the subscales of the SCHFI were 62.68 ± 22.74, 64.71 ± 22.63, and 78.24 ± 24.71 for the maintenance, monitoring, and management scales respectively indicating low self‐care maintenance and monitoring scores but a moderate self‐care management score. The mean PHQ‐9 score was 13.76 ± 6.10 reflecting moderate depression, and when categorizing each participant based on the scale categories, it was noted that the majority had moderate to moderately severe depression [28%] as indicated in Table 3. Finally, the MLHFQ mean score was 56.07 ± 25.13 reflecting a moderate QOL scores.

**TABLE 3:** Self‐care, depression and QOL affecting resilience at the bivariate level.

Variables	Range	Total (*N* = 299, 100%)	Resilient (*n* = 153, 51.2%)	Non‐resilient (*n* = 146, 48.8)	*p*
SCHFI Maintenance*	0–100	62.68 (22.74)	72.14 (18.12)	52.77 (22.91)	0.000**
SCHFI monitoring *	0–100	64.71 (22.63)	74.79 (16.47)	54.73 (23.52)	0.000**
SCHFI management*	0–100	78.24 (24.71)	89.09 (16.48)	66.87 (26.76)	0.000**
PHQ 9*	0–27	13.76 (6.10)	13.51 (5.99)	14.03 (6.22)	0.46
PHQ‐9 categories					
None‐minimal	0–4	22 (7.4)	15 (9.8)	7 (4.8)	0.367
Mild	5–9	55 (18.4)	26 (17)	29 (19.9)	
Moderate	10–14	86 (28.8)	40 (26.1)	46 (31.5)	
Moderately severe	15–19	85 (28.4)	47 (30.7)	38 (26)	
Severe	20–27	51 (17.1)	25 (16.3)	26 (17.8)	
MLHFQ*	2‐105	56.07 (25.13)	57.64 (24.22)	55.15 (25.75)	0.58

Abbreviations: MLHFQ, minnesota living with heart failure questionnaire; PHQ‐9, patient health questionnaire; SCHFI, self‐care heart failure index.

* data presented in means and standard deviation.

** significant at less than 0.05; *p* value calculated using Pearson Chi square test.

### 3.3 Resilience

Almost half of the participants in this sample were resilient, with a mean CD‐RISC score of 58.41 ± 20.97, and the mean scores of the sub‐scales of the CD‐RISC subscales were all within the moderate range, as indicated in Table 4. In terms of group differences between those who were resilient and those who were not, it was noted that the latter group were statistically significantly more smokers and took their flu vaccine statistically significantly more than their counterparts. However, the resilient group took their COVID vaccine statistically significantly more. In terms of family caregiving, those cared for by their spouse were statistically significantly more resilient, and those cared for by their children were statistically significantly less resilient than the other groups. In terms of education, those with higher education were statistically significantly more resilient than their counterparts, as indicated in Table 1.

**TABLE 4:** Resilience subscales affecting total resilience findings.

Variables	Range	Total (*N* = 299, 100%)
RISC*		58.41 (20.97)
Hardiness*	0–28	16.77 (6.55)
Coping *	0–20	11.87 (4.50)
Adaptability/flexibility*	0–12	7.48 (2.84)
Meaningfulness/purpose*	0–16	9.81 (3.62)
Optimism*	0–8	4.69 (2.01)
Regulation of emotion and recognition*	0–8	4.40 (2.03)
Self‐efficacy*	0–8	4.58 (2.02)

Abbreviation: RISC, resilience scale.

* data presented in means and standard deviation.

In terms of the medical profile, those with having dementia were statistically significantly less resilient while those with hypertension, hyperlipidemia, a previous surgical history, and those following up with medical professionals regarding their medical health were statistically significantly more resilient than their counterparts. Additionally, those taking their ACEIs/ARBs and loop diuretics were statistically significantly more resilient than those who were not, as indicated in Table 2.

Correlation analysis between the study variables showed statistically significant correlations between multiple variables. In terms of the main outcome, resilience was found to be statistically significantly negatively correlated with depression but positively correlated with self‐care maintenance, monitoring, and management. Although not significant, resilience and QOL were negatively correlated. On the other hand, when evaluating the correlations between the other study variables, it was noted that depression was statistically significantly positively correlated with QOL, which in turn was correlated with self‐care maintenance, monitoring, and management. These details are presented in Table 5.

**TABLE 5:** Correlation analysis between depression, quality of life, resilience and self‐care.

Variables	PHQ‐9	MLHFQ	RISC	SCHFI maintenance	SCHFI monitoring	SCHFI management
PHQ‐9		*r* = 0.470, *p* = 0.000*	r = −0.163, *p* = 0.005*	*r* = 0.040, *p* = 0.48	*r* = 0.065, *p* = 0.36	*r* = 0.087, *p* = 0.13
MLHFQ	*r* = 0.470, *p* = 0.000*		*r* = −0.055, *p* = 0.53	*r* = 0.233, *p* = 0008*	*r* = 0.284, 0.001*	*r* = 0.255, *p* = 0.003*
RISC	*r* = −0.163, *p* = 0.005*	*r* = −0.055, *p* = 0.53		*r* = 0.540, *p* = 0.000*	*r* = 0.475, *p* = 0.000*	*r* = 0.533, *p* = 0.000*
SCHFI maintenance	*r* = 0.040, *p* = 0.48	*r* = 0.233, *p* = 0008*	*r* = 0.540, *p* = 0.000*		*r* = 0.756, *p* = 0.000*	*r* = 0.720, *p* = 0.000*
SCHFI monitoring	*r* = 0.065, *p* = 0.36	*r* = 0.284, 0.001*	*r* = 0.475, *p* = 0.000*	*r* = 0.756, *p* = 0.000*		*r* = 0.742, *p* = 0.000*
SCHFI management	*r* = 0.087, *p* = 0.13	*r* = 0.255, *p* = 0.003*	*r* = 0.533, *p* = 0.000*	*r* = 0.720, *p* = 0.000*	*r* = 0.742, *p* = 0.000*	

Abbreviations: MLHFQ, minnesota living with heart failure questionnaire; PHQ‐9, patient health questionnaire; RISC, resilience scale; SCHFI, self‐care heart failure index.

* significant at less than 0.05.

### 3.4 Resilience Among the Geriatric Sample

Similar results were yielded for those aged more than 64 years in the study population where vaccination (flu and COVID) were undertaken more among those who were resilient. However, the direction of significance varied for the flu vaccine as those who were more resilient in the older population were more likely to be vaccinated than their counterparts (*n* = 48, 71.6% vs. *n* = 19, 28.4%; *p* < 0.001). The same direction was noted for COVID vaccination among the older and the younger subsamples. Dementia was significantly more common among those who were non‐resilient in the older adult sample, similar to the findings in the younger sample of the current study. Additionally, those who had more than secondary education were significantly more likely to be resilient than those who did not reach this level of education among the older adult sample of the study (*n* = 49, 68.1% vs. *n* = 23, 31.9%; *p* < 0.001) respectively. In terms of perceived medication adherence, it was noted that those who perceived themselves as adherent to their prescribed medications were significantly more likely to be resilient than those with poor of adherence (*n* = 65, 60.7% vs. *n* = 42, 39.3%; *p* < 0.001) respectively.

### 3.5 Multivariate Analysis of Resilience and Its Predictors

In the multiple linear regression model, self‐care maintenance, self‐care management, gender, and loop diuretic use were statistically significant predictors of resilience. The overall model was significant, *F* (4, 287) = 40.478, *p* < 0.001, and explained 36.1% of the variance in resilience scores, with an adjusted *R* ^2^ of 0.352. Higher self‐care maintenance scores (*B* = 0.248, 95% CI [0.122, 0.373], *p* < 0.001) and higher self‐care management scores (*B* = 0.282, 95% CI [0.167, 0.397], *p* < 0.001) were associated with higher resilience. Gender was also significant; because gender was coded as 0 = male and 1 = female, the negative coefficient indicates that female participants had lower resilience scores than male participants after adjustment for the other variables in the model (*B* = −4.250, 95% CI [−8.225, −0.275], *p* = 0.036). Participants using loop diuretics had higher resilience scores than those not using loop diuretics (*B* = 8.185, 95% CI [3.569, 12.800], *p* < 0.001). Collinearity diagnostics were acceptable, with VIF values ranging from 1.026 to 2.140. The details of the regression analysis are presented in Table 6.

**TABLE 6:** Regression analysis of factors affecting resilience.

Model	Adjusted R square	Durbin–Watso*n*	Unstandardized coefficients		Standard‐ized	*t*	Sig.	95% confidence interval		Collinearity statistics	
			B	Std. error	Beta			Lower bound	Upper bound	Tolerance	VIF
Constant	0.352	1.586	16.420	3.646		4.504	< 0.001*	9.245	23.596		
SCHFI maintenance			0.248	0.064	0.269	3.894	< 0.001*	0.122	0.373	0.467	2.140
SCHFI management			0.282	0.058	0.334	4.843	< 0.001*	0.167	0.397	0.469	2.132
Gender			−4.250	2.019	−0.101	−2.105	0.036*	−8.225	−0.275	0.974	1.026
Loop diuretics			8.185	2.345	0.167	3.490	< 0.001*	3.569	12.800	0.969	1.032

Abbreviations: SCHFI, self‐care heart failure index; SE, standard error; VIF, variance inflation factor.

* significant at less than 0.05.

## 4 Discussion

This study aimed to assess resilience in patients with HF in Lebanon, prompted by prior studies that reported higher‐than‐expected QOL scores within the same population (Deek and Massouh 2024; Zahwe et al. 2020). QOL scores among patients with HF nearly doubled between 2018 and 2022; however, they remained relatively stable through the period of this study conducted in 2024 and 2025. The major sociopolitical and public health disruptions in Lebanon began in 2019 and continued throughout 2020 following the Beirut blast, then settled down after 2021 when life following the COVID‐19 pandemic was returning to normal (Gostin 2022). These changes are thought to have affected the fluctuation of QOL scores over the years.

Focusing on the main outcome of interest, the moderate resilience score was comparable to that of a study conducted in Iraq (Hayef et al. 2024), likely due to the common social and political challenges and instabilities. An important finding in terms of this outcome, albeit statistically non‐significant, was the negative correlation with QOL. This finding contrasts with several studies conducted among patients facing various health challenges (Aldhahi et al. 2021; Brinkhof et al. 2021), including those with HF in a similar context in Egypt (Hassan Sherief et al. 2024). Similar results demonstrating a positive association between resilience and quality of life have also been reported in studies involving general populations not specifically focused on health‐related outcomes (Noreen et al. 2021). To date, no studies—apart from the present one—have documented a negative correlation between these two variables. Notably, none of the existing literature appears to address populations exposed to the compounded burden of ongoing health, political, social, and economic crises, as is the case in Lebanon. This discrepancy may be attributed to the persistent instability affecting the Lebanese society and the economy at multiple levels. Nonetheless, these findings warrant deeper investigation and underscore the need for further analysis and prospective evaluation.

Self‐care scores in this study are low, consistent with prior research in this population (Deek et al. 2017; Massouh et al. 2020), with some studies showing slightly higher scores possibly attributable to better socioeconomic and educational profiles within their specific sample. Notably, while self‐care maintenance and monitoring scores remained low in the current study, the self‐care management subscale demonstrated a significant improvement. In contrast to previous findings where self‐care management scores were among the lowest (47.11 ± 23.30) as reported by Deek et al. (2017)—the current sample exceeded the standard cut‐off of 70. In fact, self‐care management was the only SCHFI subscale to surpass the cut‐off in this study. This shift may reflect a growing sense of self‐reliance and independence in disease management within the Lebanese population, likely driven by the persistent sociopolitical and economic challenges that have compelled individuals to assume more active roles in their own care. This comes despite the lack of adequate/complete discharge education provided, as previously reported (Deek et al. 2020).

Another interesting controversy was the correlation between resilience and self‐care across the three studies' subscales which was shown to be significant in the current study, confirmed in the regression analysis and consistent with the literature. However, the direction of correlation was different. A study conducted in Iraq showed a negative correlation between the two studied variables (Hayef et al. 2024), while a significantly positive correlation was seen in the current study. This positive correlation was confirmed by another study that showed significant findings at the bivariate level; however, the significance was lost in the multivariate analysis (van Rijn et al. 2023). Other regression models that looked into resilience in patients with HF found sociodemographic and psychological variables to impact this outcome (Wang et al. 2022). While gender was included in the model of the current study, psychological factors did not impact this outcome. These findings qualify for further future analysis of differences between the settings, populations and challenges.

### 4.1 Interprofessional Collaborations in Gerontological Nursing

Considering the findings in terms of vaccination, education levels, and medical profile among the geriatric population, clinical and educational interventions can be undertaken to improve patient outcomes. There is also potential for interprofessional collaborative interventions targeting outcomes such as length of hospital stay, mortality, and readmission rate among others (De Gans et al. 2023). This was previously seen when interventions included care coordination, continuous monitoring and follow‐up, community‐based interventions, and care in intensive collaboration wards (De Gans et al. 2023; Zwarenstein et al. 2009). Finally, nursing interventions were seen to improve resilience levels among older adults in Arab communities similar to the current study setting (Badawy et al. 2024).

Despite the findings of the current study, some limitations warrant the explanation of these results with caution. This is due to the controversies found in the literature with the findings of the current study and the large standard deviations yielded in some variables. These could be due to the possible social desirability bias associated with self‐reported questionnaires. Additionally, the cross‐sectional approach prevented the establishment of a concrete relationship between the variables, which should be evaluated in the future. Finally, the convenience sampling method could have produced a sample with weighted characteristics, thus excluding a sample of patients with unique features that might have affected the outcome. Prospective approaches with random sampling would help to overcome these limitations in future studies. Despite these limitations, the findings of the current study shed light on some unique features of the Lebanese society that can be generalized to Lebanese diasporas in terms of assessment and management.

## 5 Conclusion

The findings of the current study highlight distinctive patterns that may inform the design of targeted interventions aimed at enhancing resilience, promoting effective self‐care behaviours, and improving overall quality of life among patients with HF. Moreover, examining the sociodemographic, medical, social, and psychological characteristics of this population is essential for informing evidence‐based nursing policies and care practices tailored to the needs of individuals living with HF.

## Author Contributions

**Hiba Deek:** conceptualization, methodology, software, data curation, investigation, validation, formal analysis, supervision, visualization, project administration, resources, writing – original draft, writing – review and editing. **Angela R. Massouh:** conceptualization, methodology, data curation, investigation, formal analysis, writing – original draft. **Wassim Shatila:** methodology, investigation, writing – original draft.

## Funding

The authors have nothing to report.

## Disclosure

Declaration of Generative AI and AI‐Assisted Technologies in the Manuscript Preparation Process: During revision, AI‐assisted language support was used to improve clarity, organization, and readability. The authors reviewed, edited, and verified all content and take full responsibility for the final manuscript.

*Declaration*: The authors have checked to make sure that our submission conforms as applicable to the Journal's statistical guidelines.

*Statistical Declaration*: The statistical analysis plan was developed and reviewed by the study team. Descriptive, bivariate, and multivariable analyses were conducted using IBM SPSS Statistics version 29.0. Statistical significance was set at *p* < 0.05 with 95% confidence intervals reported where applicable.

*Reporting guidelines*: The paper is reported following the STROBE checklist (Data S1).

*Clinical Resources*: https://theheartfoundation.org/heart‐health‐tools‐and‐resources/; https://myglobalheart.org/resources/.

## Ethics Statement

Ethical approval was secured from the Institutional Review Board of the Beirut Arab University [2023‐H‐0156‐HS‐R‐0550].

## Consent

After ensuring that participants understood the study goal, a signed informed consent was secured, and data were collected by a trained research team.

## Conflicts of Interest

The authors declare no conflicts of interest.

## Supporting information


**Data S1:** STROBE checklist.

## Data Availability

The data that support the findings of this study are available on request from the corresponding author. The data are not publicly available due to privacy or ethical restrictions.
